# Improving access to community-based pulmonary rehabilitation: 3R protocol for real-world settings with cost-benefit analysis

**DOI:** 10.1186/s12889-019-7045-1

**Published:** 2019-05-31

**Authors:** Alda Marques, Cristina Jácome, Patrícia Rebelo, Cátia Paixão, Ana Oliveira, Joana Cruz, Célia Freitas, Marília Rua, Helena Loureiro, Cristina Peguinho, Fábio Marques, Adriana Simões, Madalena Santos, Paula Martins, Alexandra André, Sílvia De Francesco, Vitória Martins, Dina Brooks, Paula Simão

**Affiliations:** 1 Respiratory Research and Rehabilitation Laboratory (Lab3R), School of Health Sciences (ESSUA), University of Aveiro, Agras do Crasto - Campus Universitário de Santiago, Edifício 30, 3810-193 Aveiro, Portugal; 2 Institute of Biomedicine (iBiMED), University of Aveiro, Agras do Crasto - Campus Universitário de Santiago, Edifício 30, 3810-193 Aveiro, Portugal; 3 CINTESIS – Center for Health Technology and Services Research, Faculty of Medicine, University of Porto, Porto, Portugal; 4 School of Health Sciences (ESSLei), Center for Innovative Care and Health Technology (ciTechCare), Polytechnic Institute of Leiria, Leiria, Portugal; 5 Research Centre on Didactics and Technology in the Education of Trainers (CIDTFF), University of Aveiro, Aveiro, Portugal; 6 Higher Institute for Accountancy and Administration (ISCA-UA), University of Aveiro, Aveiro, Portugal; 7 ESTGA - Águeda School of Technology and Management, Águeda, Portugal; 8 Câmara Municipal de Aveiro, Aveiro, Portugal; 9 Câmara Municipal de Mira, Mira, Portugal; 10 College of Health Technology of Coimbra (ESTeSC), Polytechnic Institute of Coimbra, Coimbra, Portugal; 11 IEETA - Institute of Electronics and Informatics Engineering of Aveiro, Aveiro, Portugal; 12 Pulmonology Department, Hospital Distrital da Figueira da Foz, Figueira da Foz, Portugal; 13 Respiratory Medicine, West Park Healthcare Centre, and University of Toronto, Toronto, Canada; 14 School of Rehabilitation Sciences, Faculty of Health Sciences, McMaster University, Hamilton, Canada; 15 Pulmonology Department, Unidade Local de Saúde de Matosinhos, Matosinhos, Portugal

**Keywords:** Exercise training, Education and psychosocial support, Chronic respiratory diseases, Primary healthcare, Cost-benefit

## Abstract

**Background:**

Pulmonary rehabilitation (PR) has demonstrated patients’ physiological and psychosocial improvements, symptoms reduction and health-economic benefits whilst enhances the ability of the whole family to adjust to illness. However, PR remains highly inaccessible due to lack of awareness of its benefits, poor referral and availability mostly in hospitals. Novel models of PR delivery are needed to enhance its implementation while maintaining cost-efficiency. We aim to implement an innovative community-based PR programme and assess its cost-benefit.

**Methods:**

A 12-week community-based PR will be implemented in primary healthcare centres where programmes are not available. Healthcare professionals will be trained. 73 patients with CRD and their caregivers (dyads patient-caregivers) will compose the experimental group. The control group will include dyads age- and disease-matched willing to collaborate in data collection but not in PR. Patients/family-centred outcomes will be dyspnoea (modified Medical Research Council Questionnaire), fatigue (Checklist of individual strength and Functional assessment of chronic illness therapy – fatigue), cough and sputum (Leicester cough questionnaire and Cough and sputum assessment questionnaire), impact of the disease (COPD Assessment Test), emotional state (The Hospital Anxiety and Depression Scale), number of exacerbations, healthcare utilisation, health-related quality of life and family adaptability/cohesion (Family Adaptation and Cohesion Scale). Other clinical outcomes will be peripheral (biceps and quadriceps-hand held dynamometer, 1 or 10 repetition-maximum) and respiratory (maximal inspiratory and expiratory pressures) muscle strength, muscle thickness and cross sectional area (biceps brachialis, rectus femoris and diaphragm-ultrasound imaging), exercise capacity (six-minute walk test and one-minute sit to stand test), balance (brief-balance evaluation systems test) and physical activity (accelerometer). Data will be collected at baseline, at 12 weeks, at 3- and 6-months post-PR.

Changes in the outcome measures will be compared between groups, after multivariate adjustment for possible confounders, and effect sizes will be calculated. A cost-benefit analysis will be conducted.

**Discussion:**

This study will enhance patients access to PR, by training healthcare professionals in the local primary healthcare centres to conduct such programmes and actively involving caregivers. The cost-benefit analysis of this intervention will provide an evidence-based insight into the economic benefit of community-based PR in chronic respiratory diseases.

**Trial registration:**

The trial was registered in the ClinicalTrials.gov U.S. National Library of Medicine, on 10th January, 2019 (registration number: NCT03799666).

**Electronic supplementary material:**

The online version of this article (10.1186/s12889-019-7045-1) contains supplementary material, which is available to authorized users.

## Background

Respiratory diseases represent five of the thirty most common causes of death worldwide [1, 2] and account for more than 10% of all disability-adjusted life-years [1]. It is known that these conditions impact on economic and social systems [3] but most importantly, cause enormous challenges for individuals and respective families. These patients experience disabling symptoms (including dyspnoea, fatigue, cough, sputum, anxiety and depression), limitation in their activities of daily life and social/family interactions, exercise intolerance, low physical activity levels and impairments in their quality of life [4–19]. Caregivers provide invaluable support to these patients, i.e., emotional/spiritual (e.g., someone to whom they can talk to), physical/practical (e.g., dressing, mobility assistance, medication check, overnight vigilance) and social and financial [20, 21]. Although they report positive experiences, negative impacts and specific needs directly related to their role have also been widely acknowledged [20–25]. However, very little has been done to support caregivers and there is lack of interventional studies [20, 21]. Pulmonary rehabilitation (PR) has been proposed as a possible response [21, 23, 26] since it could actively involve the family system within care delivery [27].

PR is a comprehensive intervention, which includes exercise training, education and behaviour change, to improve patients’ physical and psychological wellbeing and to promote their long-term adherence to health-enhancing behaviours [28]. In patients with chronic respiratory diseases (CRD) (chronic obstructive pulmonary disease [COPD], interstitial lung disease, bronchiectasis, cystic fibrosis, asthma, pulmonary hypertension, lung cancer), PR has demonstrated physiological and psychosocial improvements, symptoms reduction and health economic benefits [10, 29–37]. It has also shown to enhance the ability of the whole family to cope and psychosocially adjust to illness [27]. Given its benefits, PR has been proposed to be part of the standard care offered to patients with CRD [10, 28]. However, it continues to be highly inaccessible [10]. Patients lack awareness of its benefits, there is poor referral from healthcare professionals and programmes are mostly hospital-based and directed at patients with COPD at advanced stages [28, 38]. Therefore, it has been acknowledged that several steps are needed to increase access to PR, such as: i) enhancing accessibility of the existing programmes; ii) increasing the number of programmes especially in the community; iii) developing and validating novel models to deliver sustainable PR; iv) increasing PR reimbursement and payer acceptance; v) exploring its cost-benefit; vi) promoting maintenance of long-term results and vii) identifying those who should be prioritised [28, 38]. This study will address some of these steps. It is hypothesised that community-based PR, directed to patients with several CRD, at all grades of the disease, and involving different local stakeholders (i.e., healthcare professionals, patients, caregivers, decision-makers and the local community) may turn PR more accessible, sustainable and cost-effective.

## Methods

### Aims

The main goal of this project is to implement an innovative community-based PR programme and assess its cost-benefit. The primary objectives are to:

1.1. Investigate the short- and medium-term effects of community-based PR programmes, implemented with minimal resources and with local healthcare professionals, in patients and caregiver’s health-related quality of life;
1.2. Investigate the cost-benefit of the community-based PR programmes on acute exacerbations and healthcare utilisation, to determine whether the intrinsic perceived social value of such programmes have identifiable benefits and, consequently, measurable economic value for society.

The following secondary objectives will be addressed:

2.1 To explore the short- and medium-term effects of community-based PR programmes on patients and caregivers’ symptoms, impact of the disease, family adaptability/cohesion, peripheral and respiratory muscle strength, exercise capacity, balance and physical activity.
2.2 To explore differences between patients and caregivers’ who participate and those who did not participate in community-based PR programmes in relation to dyspnoea, fatigue, cough, impact of the disease, emotional state, health-related quality of life, number of exacerbations, healthcare utilization, family adaptability/cohesion, peripheral and respiratory muscle strength and thickness, exercise capacity, balance and physical activity.
2.3 To explore associations between patients and caregivers’ outcomes of dyspnoea, fatigue, cough, impact of the disease, emotional state, number of exacerbations, healthcare utilisation, family adaptability/cohesion, peripheral and respiratory muscle strength and thickness, exercise capacity, balance and physical activity, pre-post community-based PR programmes.
2.4 To investigate the short-term effects of community-based PR programmes on patients’ peripheral muscle (quadriceps and biceps) and diaphragm thickness, cross sectional area, echointensity and motion measured resorting to ultrasound imaging.

### Study design, setting and recruitment

A real-world non-randomised controlled study conducted in the community has been designed. Coordinators of primary healthcare centres will be approached in an arranged meeting to explain the study and those interested to participate will be asked to identify the multidisciplinary team available in their primary healthcare centre. Indication for the minimum staff required will be provided, i.e., a physician, a physiotherapist and a nurse; however, emphasis on the importance to include other professionals (e.g., nutritionist, psychologist, social worker), if available, will be given, following the international recommendations [28, 39, 40]. General practitioners and/or pulmonologists will identify eligible stable patients with a CRD (e.g., COPD, asthma, asthma-COPD overlap, interstitial lung disease, etc.) and their caregivers, and explain the study. Only patients and caregivers with interest in participating will be contacted by the researchers. A meeting will then be arranged to provide written and oral information about the study and collect the informed consent (Additional file 1). The researchers also explained that all data would be kept in databases password protected, using codes and their names would never be disclosed, ensuring the confidentiality and anonymity of all data. Two groups will be composed, experimental (EG) and control (CG). The EG will include patients and caregivers wanting to participate in a 12-week community-based PR programme and the CG will include those age- and disease-matched willing to collaborate in data collection but not in the PR programme. Recruitment started in January 2019, with final data collection expected to be completed in December 2019.

### Patient and public involvement

Patients and public were not directly involved in the study design or recruitment in this study however, authors were informed about their needs, preferences and expectations, as well as on the outcomes by recent published papers of the research team, and took those into consideration when designing the study [41, 42].

### Eligibility criteria

Patients will be eligible if they are diagnosed with a CRD [43, 44] and clinically stable in the previous month (i.e., no hospital admissions, exacerbations or changes in medication for the cardiorespiratory system). Exclusion criteria will be the presence of any clinical condition that can preclude participants of being involved in the community-based PR programme, such as, signs of cognitive impairment (e.g. dementia) or presence of a significant cardiovascular (e.g. symptomatic ischaemic cardiac disease), neurological (e.g. neuromuscular dystrophy disease) or musculoskeletal disease (e.g. important kyphoscoliosis). Caregivers will be included if they are: ≥18 years old and living with or providing physical/supportive care to the patient, and excluded if they are unable to cooperate.

### Data collection

Sociodemographic (age, sex, educational level, marital and working status), anthropometric (height and weight to calculate the body mass index) and general clinical data (long-term oxygen, non-invasive ventilation, medical history, comorbidities to calculate the Charlson Comorbidity Index [45] and medication) will first be collected with a structured questionnaire to characterise the sample and will be followed by a lung function test with spirometry [46].

The primary outcome measure will be health-related quality of life, assessed with the St. George Respiratory Questionnaire (SGRQ) [47] in patients and with the World Health Organization Quality of Life Bref Questionnaire [48, 49] in caregivers. The number of acute exacerbations, healthcare utilisation costs and collateral costs (e.g. transport costs, work absence costs, sickness benefits) will also be collected to conduct the cost-benefit analysis [50].

In addition, the following secondary outcomes will be assessed: symptoms of dyspnoea (modified Medical Research Council Questionnaire [mMRC]) [51], fatigue (Checklist of individual strength [CIS-20]) [52] and Functional assessment of chronic illness therapy – fatigue [FACIT-F]) [53, 54], cough and sputum (Leicester cough questionnaire [LCQ] [55] and Cough and sputum assessment questionnaire [CASA-Q]) [56], impact of the disease (COPD Assessment Test [CAT]) [57, 58], emotional status (The Hospital Anxiety and Depression Scale [HADS]) [59, 60], Family Adaptation and Cohesion Scales [FACES-IV]) [61, 62], peripheral (biceps and quadriceps with the hand held dynamometer, 1 [1-RM] or 10 [10-RM] repetition-maximum) [63, 64] and inspiratory and expiratory muscle strength (respiratory pressure meter) [65], exercise capacity (six-minute walk test [6MWT] and one-minute sit to stand test [1-min STS]) [66, 67], balance (brief-balance evaluation systems test [Brief-BESTest]) [68] and physical activity (accelerometer) [14]. Peripheral muscle (rectus femoris and biceps brachialli) and diaphragm thickness, cross sectional area [69, 70] and echointensity [71], excursion and M-Mode Index of Obstruction (MIO) [72–74], will be measured with ultrasound images – ImageJ and Matlab software. Global rating of change scale [75] for fatigue, cough, sputum, peripheral and respiratory muscle strength and balance will also be collected.

Data will be collected from patients and caregivers at baseline, at 12 weeks and at 3- and 6-months post-PR. Ultrasound imaging will only be collected at baseline and at 12 weeks in patients from the experimental group. Additional file 2 provides an overview of enrolment, intervention and outcomes to be assessed in each time point.

### Intervention

A 12-week community-based PR programme will be implemented in primary healthcare centre with minimal resources (i.e., pulse oximeters, blood pressure monitors, modified Borg scales, chairs, stairs, corridors, free weights built with bottles with sand when others are not available, elastic bands and cushions). Healthcare professionals from each primary healthcare centres will receive two sessions of training, of three to 4 hours each, prior to starting the programme. These sessions will focus on how to assess patients’ comprehensively, main modules to approach in the education and psychosocial components and importance of involving a multidisciplinary team. Physiotherapists will also revise principles of exercise safety, prescription and patients’ monitoring. The PR programme will then be implemented in collaboration with members of the research team, who are highly experienced professionals in running PR. When the programmes finish, local healthcare professionals will continue to implement programmes by themselves and the research team will be available for assistance and clarification of doubts by phone.

The programme will be composed of pre/post PR comprehensive assessment of patients and caregivers, two weekly sessions of exercise training and one session of education and psychosocial support every other week. Caregivers of the EG will be invited to participate in these sessions that will be delivered in group, but personalised to each patient/caregiver.

**Fig. 1:**
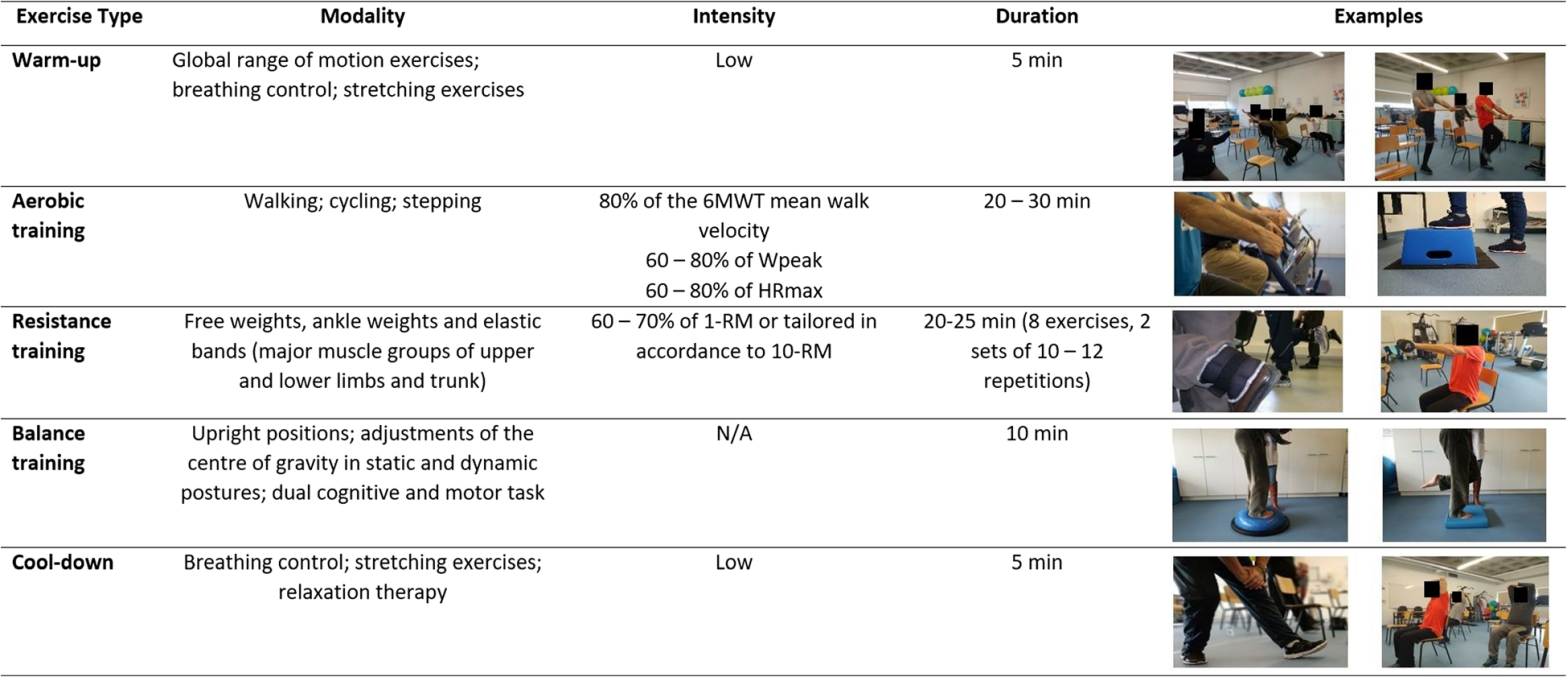
Community-based pulmonary rehabilitation – exercise training component. 6MWT – 6-min walk test; Wpeak - work peak; HRmax – maximum heart rate; 1-RM – 1 repetition maximum; 10-RM – 10 repetition maximum; N/A – not applicable. Consent from participants was obtained to publish the data

Each exercise session will last between 60 and 75 min and will be delivered by an experienced physiotherapist in accordance with the international guidelines [28], i.e., it will include warm up, aerobic, resistance and balance training and a cool down period. Furthermore, inspiratory muscle training [65] will be provided if maximal inspiratory pressure is < 80 cmH_2_O [76]. Heart rate and oxygen saturation (with a pulse oximeter) and perceived dyspnoea and fatigue (with the modified Borg scale) [77], will be monitored throughout the sessions. Intensity of the aerobic and resistance training will be individually prescribed using the 6MWT [66] and the 1-RM or 10-RM methods [64] (considering the availability of the local equipment), respectively. After a 5 min. Warm-up period (range-of-motion, stretching, low-intensity aerobic exercises and breathing techniques), aerobic training will be conducted in corridors and stairs/steps or in cycloergometers or treadmills if available in the facilities, for 20–30 min at 80% of the average speed achieved during the 6-MWT, or 60 to 80% of their work peak, or 60 to 80% of maximum heart rate [40, 78]. Resistance training will consist of 8 exercises of the major upper and lower limb muscle groups, at 60 to 70% of 1-RM or tailored in accordance to the 10-RM, using free weights and ankle weights for 20–25 min [79]. A balance training component will follow for 10 min with exercises for the six subsystems of balance control [80] and then the programme will finish with a 5 min. Cool down period. During the PR programme, progression in the training intensity will be tailored according to the perceived dyspnoea and fatigue (4–6 in the modified Borg scale). A detailed description of the exercise training component can be found in Fig. 1.

**Fig. 2:**
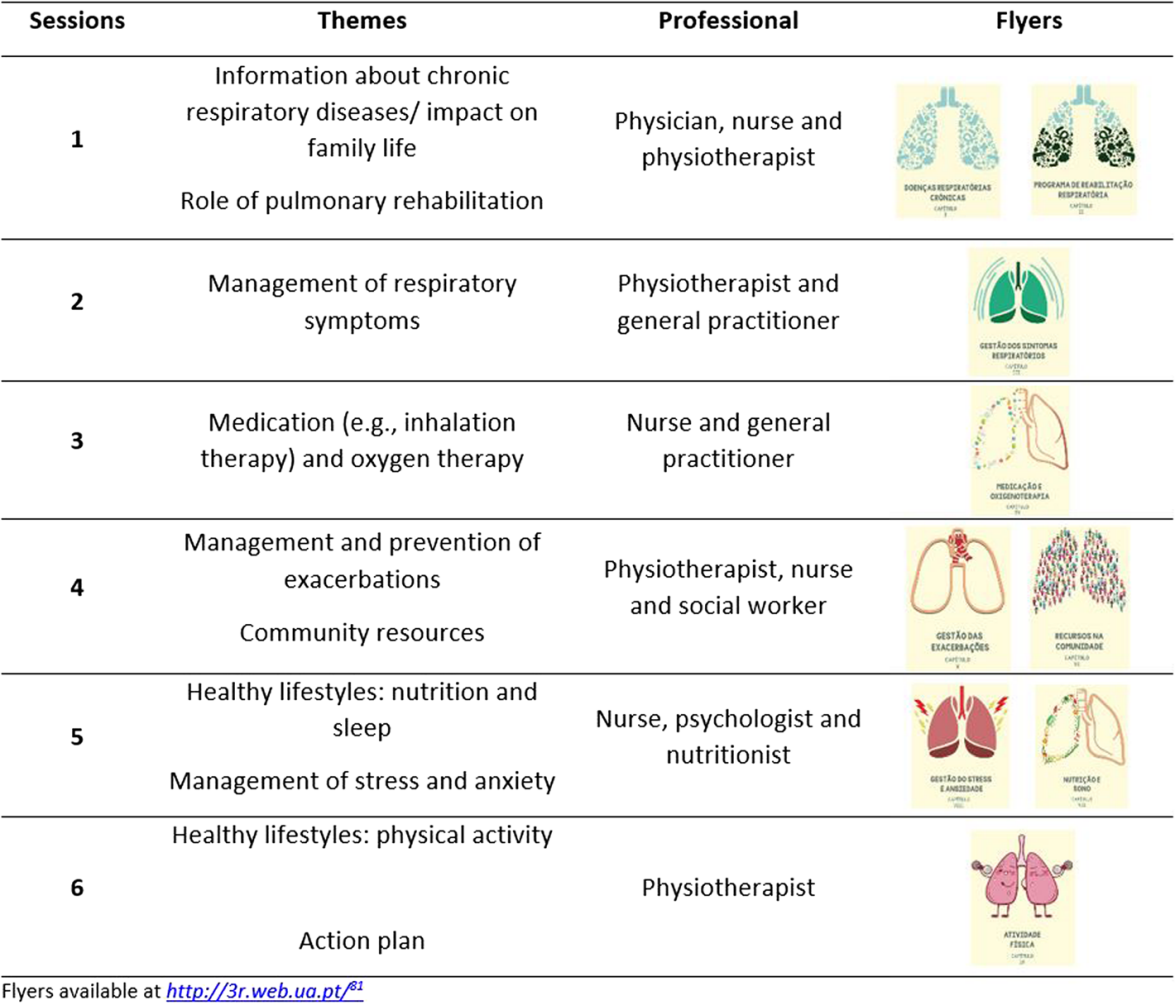
Community-based pulmonary rehabilitation – education and psychosocial component

Each education and psychosocial session will last approximately 90 min. Six sessions will be conducted by a multidisciplinary team. Based on the literature and previous experience, it is anticipated that some themes will need to be addressed [42, 81–84] however, the component will be tailored to patients and caregivers’ needs. A detailed description of these sessions can be found in Fig. 2.

**Fig. 3:**
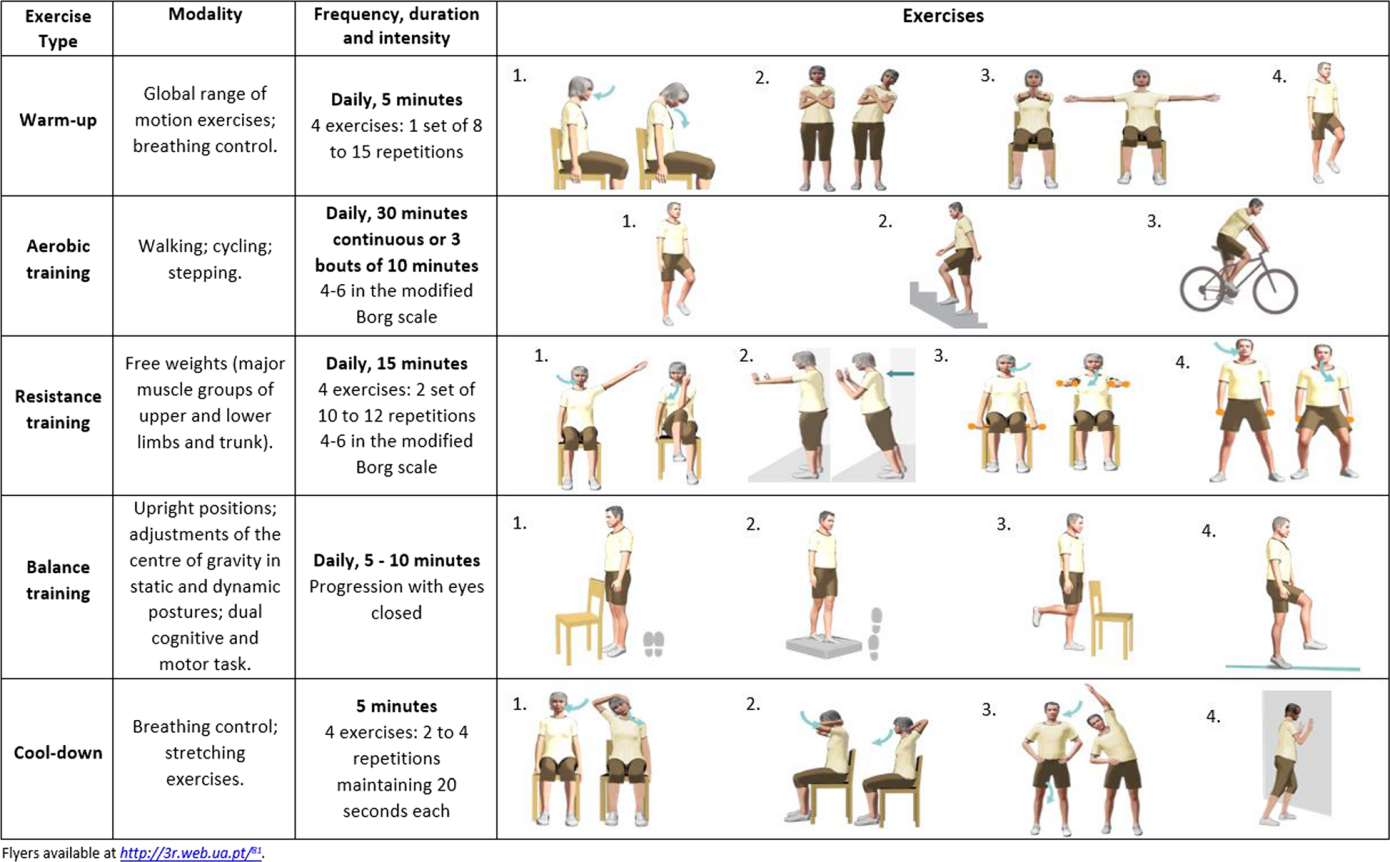
Exercise options and physical activity recommendations for patients to perform at home

Patients will also receive recommendations for home training to complete on the additional days: i) aerobic, resistance and balance exercises and general health counselling to perform ⩾30 min of moderate physical activity ⩾5 days per week [85]. A leaflet with some exercise options and physical activity guidance will be provided (Fig. 3). Moreover, they will sign a physical activity contract, which consists of encouraging self-efficacy and establishing individualised goal setting [86]. For this purpose, the final goal of steps average count will be negotiated with the patients, accordingly to their baseline, i.e., those with > 9000 steps/day - maintain or increase steps/day; those with ≥6000 and < 9000 steps/day - reach 9000 steps/day and in those with < 6000 steps - increase 3000 steps/day by the end of the programme [87]. Furthermore, the physiotherapist will provide weekly-feedback to encourage patients to increase 562 steps every week [88]. Participants will receive a pedometer and a physical activity diary, to register their daily steps.

The CG will continue to receive the standard care from the primary healthcare team.

### Sample size

A sample size estimation with 80% power at 5% significance was calculated to detect significant differences in patients’ health-related quality of life assessed with the SGRQ. The pre-post score achieved in the intervention group of a PR in COPD integrating family-based education and psychosocial support was used (Pre 37.9 ± 18.2 vs. Post 31.4 ± 18.7, *p* < 0.001) [27], resulting in a total sample size of 73 participants. In PR programmes, dropout rates are approximately 20 to 40% [89, 90]. Therefore, 102 participants will be recruited. Sample size calculation was performed using G*Power 3.1.3 (Universität Düsseldorf, Düsseldorf, Germany).

### Data management and statistical analysis

All variables will be processed in IBM SPSS or MS Excel software. Descriptive statistics, including frequencies, means and standard deviations, medians and interquartile ranges, will be used as appropriate. Short- and medium-term effects of the community-based PR programmes will be verified using paired t-test or Wilcoxon signed-rank tests, accordingly to data normality. Mean scores of continuous variables will be compared between patients and their caregivers using independent samples t-test or Mann Whitney U test, depending on the variable distribution. Pearson correlation coefficient [91] and Bland and Altman plots [92] for continuous variables and Cohen’s k [93] for categorical variables, will be applied to study the relationship and agreement between patients and caregivers. Relationships between variables will be explored with inferential, correlational and univariate/multivariate analyses in SPSS. Differences between the EG and CG at different time points will be assessed using a two-way ANOVA with repeated measures (continuous data), Kruskal-Wallis (ordinal data) and Chi-square or Fisher exact probability tests (nominal data). If missing data is found during the study follow up, the generalized estimating equation’ models will be applied. This method is an extension of generalized linear models to longitudinal data which permits the inclusion of time-dependent variables and the analysis of incomplete data (without imputing missing data), common in longitudinal health studies [94]. A significance level of 0.05 will be used.

Whenever possible, minimal clinically important differences (MCID) will be established, following the current recommendations to integrate both anchor-based and distribution-based approaches [95, 96]. The final MCID for each measure will be pooled using MetaXL 5.3 (EpiGear International, Queensland, Australia), with the input data being the MCID generated by each anchor- and distribution-based method and, when appropriate, the respective confidence interval. A quality effects model [97] will be used and anchor-based methods will weigh more than distribution methods (i.e., 2/3 against 1/3) [98].

A cost-benefit analysis will be conducted to determine the economic value of the perceived social value of community-based PR programmes. This analysis allows for the aggregation of both health and non-health benefits and costs of the PR programme and provides useful and quantifiable information [99, 100], not only for patients and health care institutions, but also for decision makers [101].

Focusing on the frequency and length of exacerbations, healthcare utilisation costs and quality of life gains, along with several indirect costs and benefits associated with PR implementation, including productivity loss of patients and caregivers who provide assistance, working, transportation costs, administrative costs, opportunity costs, sickness benefits payed to the patient or professional training and recruiting costs, three complementary data analysis will be held: cost:-effectiveness, −utility and -benefit.

As recent studies on COPD have demonstrated, it is suitable for cost-effectiveness analysis to rest on the ratio *incremental cost per exacerbation/healthcare utilisation avoidance* [102] aiming to compare relative costs and outcomes of PR. Then, to assess the expected impact on patients’ health-related quality of life, cost-utility analysis computes the measures of quality adjusted life years (QALY) [103] and healthy years equivalents (HYEs) [104] and determines the incremental cost per QALY/HYE gained. Finally, to account for the monetary translation of positive and negative effects of community-based PR programmes, cost-benefit analysis will be performed by determining the measures of net present monetary benefit and economic internal rate of return [105], along with a sensitivity analysis to control for the uncertainty on the assessment of costs and benefits associated to the community-based PR programmes [106, 107].

## Discussion

This real-world study will focus on enhancing patients’ access to PR by implementing it within their community, with the staff available in local primary healthcare centres and actively involving informal caregivers. It will also compute a measure of cost-benefit of implementing this intervention in the community. This information is needed for advocating the wide dissemination of PR across the world, actively involve and train caregivers and more healthcare professionals in the disease management of patients with CRD.

This study differs from others, by implementing a fundamental intervention for all patients with CRD, at different development stage of their disease, within the community, near their homes and where it is known that this intervention is lacking [38]. Patients with chronic respiratory conditions have been accessing to PR mainly in hospitals where constrains regarding availability, transportation, funding and referral exist [28, 38, 108]. Although community-based PR in patients with COPD has been found to be effective for health-related quality of life [35, 36] and exercise capacity [36], caution interpreting these results is needed as relatively few studies exist and they were conducted in several settings (home, primary healthcare centres, a mixture of more than one setting) with disparities in the structure and components of the intervention, hindering strong conclusions regarding the effects of community-based PR. In other CRD, comparisons across settings are not even possible due to the lack of studies [29, 30, 33]. Recently, a community-based PR programme conducted in a non-healthcare facility with patients with several CRD demonstrated positive effects on patients’ exercise capacity, health-related quality of life, and a reduction in respiratory-related hospital admissions in the 12 months following the programme [108]. However, primary healthcare centres are embedded within the community, have a multidisciplinary team available and most patients and their families have their routine follow-ups in these facilities. Therefore, these community healthcare infrastructures might be ideal candidates to enhance patients’ access to PR but, staff often lacks training and equipment is scarce. This study proposes to deliver a highly structured training to available primary healthcare staff where PR is non-existent, supervise the staff during one programme delivery, using minimal resources, and guide them on the development or acquisition of equipment. This will increase the PR response to patients with chronic respiratory conditions but, will also raise awareness in more healthcare professionals for this intervention. It is also known that caregivers are the main providers of support to these patients [20, 21] and it has been demonstrated that when they are integrated in PR programmes the whole family benefits from it [27]. Therefore, this project will be innovative by empowering healthcare professionals of health facilities where PR programmes are not available, but also by actively involving caregivers in this intervention which together is believed to improve dissemination and sustainability of PR in the community directed to patients with several CRD.

In addition, positive effects are expected to arise from the cost-benefit analysis. The need to increase awareness and knowledge of PR among rulers and decision makers has been widely identified, and an economic evaluation might be able to provide “value-for money” information and promote the dialogue among different stakeholders and consequently wider dissemination of PR programmes [10, 38].

Moreover, the longitudinal design will facilitate analysis of changes over time in a comprehensive set of measures enhancing our knowledge on patients’ evolution. This will also represent the ideal opportunity to explore a wide range of emergent ultrasound measures to assess the short-term effects of PR on the structure and motion of the diaphragm and peripheral muscles (biceps and quadriceps). Ultrasonographic assessment of the rectus femoris muscle (thickness and cross sectional area) has been found to be correlated with muscle strength [70], and some measures of diaphragmatic kinetics have been proposed as promising to study disease progression (e.g., MIO) [73, 74] and prognosis of PR outcomes (e.g. change in the diaphragmatic length of zone of apposition at functional residual capacity ΔLzapp%) [109]. Nevertheless, relatively little is yet known about the potential of the ultrasonographic assessment to assess the effects of PR on peripheral muscle (rectus femoris, biceps brachialis) and diaphragm thickness, cross sectional area [69, 70] and echointensity [71], excursion and MIO [72–74].

It is important to acknowledge the limitations of this study. Although it is intended to implement a real-world study as inclusive as possible and, therefore patients with distinct CRD will be eligible, this might poses challenges during the recruitment and implementation phases. This limitation will be minimised by clarifying and emphasising the inclusion criteria in the meetings with healthcare teams when explaining the study. Additionally, an experienced member in implementing PR programmes will be present in all sessions together with the staff of the primary healthcare centre to help with the personalised interventions. The inclusion of chronic respiratory patients may also underpower the study. To mitigate this risk, power calculations were based only on patients with COPD, since they are the most common population referred to PR programmes. Nevertheless, it will be possible to use data from this study to compute power calculations for other studies.

Another anticipated limitation is the recruitment of caregivers. Caregivers are rarely included in PR programmes therefore, they might not be aware of the possible response that PR can provide not only to patients but for the whole family. The importance of their inclusion will be discussed with patients and caregivers when explaining the study. A written information sheet highlighting the importance of their inclusion will be provided to promote their inclusion.

Moreover, acknowledging the benefits that PR has for patients with CRD, not offering PR to all eligible patients will never be considered. Therefore, the CG will be composed of only those patients who are willing to participate in data collection but do not want take part in the PR programme. This might lead to difficulties in recruiting for the CG. Nevertheless, with the permission of patients and caregivers, a telephone call will be made prior to data collection to minimise dropouts.

A follow-up period of 6 months could be too short to draw conclusions about the medium- or long-term changes in patients’ health and non-health measures, necessary for cost-benefit analysis. However, limited funding is available for the study.

Finally, obtaining cooperation of patients during the ultrasound measurements of the diaphragm might be challenging as these patients often present significant dyspnoea and fatigue levels, limiting their collaboration in the requested breathing manoeuvres.

We expect that this study will enhance the patients’ access to PR and provide an evidence-based insight into the economic benefit of community-based PR in chronic respiratory diseases, through a cost-benefit analysis.

## Additional files

Additional file 1: Participants’ informed consent. Free informed consent given to patients prior to integrate the study. (ZIP 2147 kb)

Additional file 2: 3R protocol schedule of enrolment, interventions and outcomes (adapted from original table^1^). A table with an overview of enrolment, intervention and outcomes to be assessed in each time point in the study. (DOCX 23 kb)

## Data Availability

Not applicable.

## Abbreviations

10-RM: 10 repetition-maximum
1-min STS: One-minute sit to stand test
1-RM: 1 repetition-maximum
6MWT: Six-minute walk test
Brief-BESTest: Brief-balance evaluation systems test
CASA-Q: Cough and sputum assessment questionnaire
CAT: COPD Assessment Test
CG: Control group
CIS-20: Checklist of individual strength
COPD: Chronic obstructive pulmonary disease
CRD: Chronic respiratory diseases
EG: Experimental group
FACES-IV: Family Adaptation and Cohesion Scales
FACIT-F: Functional assessment of chronic illness therapy – fatigue
HADS: The Hospital Anxiety and Depression Scale
HYES: Healthy years equivalents
LCQ: Leicester cough questionnaire
MCID: Minimal clinically important differences
MIO: M-Mode Index of Obstruction
mMRC: Modified Medical Research Council Questionnaire
PR: Pulmonary rehabilitation
QALY: Quality adjusted life years
SGRQ: St. George respiratory questionnaire
