# Supplementary material for: Improving access to community-based pulmonary rehabilitation: 3R protocol for real-world settings with cost-benefit analysis
Source: BMC Public Health. 2019 May 31;19:676. doi: 10.1186/s12889-019-7045-1 (PMC6544941; doi:10.1186/s12889-019-7045-1)
Supplement: Supplementary file 1 — Participants’ informed consent. Free informed consent given to patients prior to integrate the study. (ZIP 2147 kb) [file 12889_2019_7045_MOESM1_ESM.zip › Additional file 1_ENGR1.docx]

**Additional file 1 –** Participants’ informed consent

**Informed consent form**

**Project title: “**Revitalizing pulmonary rehabilitation (3R)”.

**Name of the principal investigator:** Doctor Alda Sofia Pires de Dias Marques

**Please read and mark with a cross (X) the following boxes.**

| 1. I confirm that I have understood all the information provided to me and that I have had the opportunity to clarify any doubt. |  |
| --- | --- |
| 1. I understand that my participation in the study is voluntary and that I am free to withdraw at any time without giving any reason. This will not affect any health or social care provided to me. |  |
| 1. I understand that all data collected during the study will be confidential and only the investigators of the study at the University of Aveiro will have access to them. Thus, I authorise them the use of these data. 2. I understand that all data collected during the study can be used for publication in scientific journals, or used in other investigations, without any breach of confidentiality. Thus, I authorise the use of the data to that purpose. 3. Therefore, I agree to participate in the study. |  |
|  |  |
|  |  |

| ________________________  Name of the participant | _________  Date | ___________________________  Signature |
| --- | --- | --- |
| ________________________  Name of the investigator | _________  Date | ___________________________  Signature |
