# Supplementary material for: Improving access to community-based pulmonary rehabilitation: 3R protocol for real-world settings with cost-benefit analysis
Source: BMC Public Health. 2019 May 31;19:676. doi: 10.1186/s12889-019-7045-1 (PMC6544941; doi:10.1186/s12889-019-7045-1)
Supplement: Supplementary file 1 — Participants’ informed consent. Free informed consent given to patients prior to integrate the study. (ZIP 2147 kb) [file 12889_2019_7045_MOESM1_ESM.zip › Additional file 1_PTR1.docx]

**Additional file 1 –** Participants’ informed consent

**Termo de Consentimento Livre e Esclarecido**

**Título do Projeto: “**Revitalizar a Reabilitação Respiratória (3R)”.

**Nome do Investigador Principal:** Prof. Doutora Alda Sofia Pires de Dias Marques

**Por favor leia e assinale com uma cruz (X) os quadrados seguintes.**

| 1. Eu confirmo que percebi a informação que me foi dada e tive a oportunidade de questionar e de me esclarecer. |  |
| --- | --- |
| 2. Eu percebo que a minha participação é voluntária e que sou livre de desistir, em qualquer altura, sem dar nenhuma explicação, sem que isso afete qualquer serviço de saúde ou qualquer outro que me é prestado. |  |
| 3. Eu compreendo que os dados recolhidos durante a investigação são confidenciais e que só os investigadores do projeto da Universidade de Aveiro têm acesso a eles. Portanto, dou autorização para que os mesmos tenham acesso a esses dados. |  |
| 4. Eu compreendo que os dados recolhidos durante o estudo podem ser utilizados para publicação em Revistas Científicas e usados noutras investigações, sem que haja qualquer quebra de confidencialidade. Portanto, dou autorização para a utilização dos dados para esses fins. |  |
| 5. Eu concordo então em participar no estudo. |  |

| ________________________  Nome da pessoa | _________  Data | ___________________________  Assinatura |
| --- | --- | --- |
| ________________________  Nome do Investigador(a) | _________  Data | ___________________________  Assinatura |
