# Supplementary material for: Improving access to community-based pulmonary rehabilitation: 3R protocol for real-world settings with cost-benefit analysis
Source: BMC Public Health. 2019 May 31;19:676. doi: 10.1186/s12889-019-7045-1 (PMC6544941; doi:10.1186/s12889-019-7045-1)
Supplement: Supplementary file 2 — 3R protocol schedule of enrolment, interventions and outcomes (adapted from original table1). A table with an overview of enrolment, intervention and outcomes to be assessed in each time point in the study. (DOCX 23 kb) [file 12889_2019_7045_MOESM2_ESM.docx]

Additional file 2 - 3R protocol schedule of enrolment, interventions and outcomes (adapted from original table^1^)

|  | **STUDY PERIOD** | | | | |
| --- | --- | --- | --- | --- | --- |
|  | **Enrolment** | **Baseline** | **After 12-week PR programme** | **3-months post-PR** | **6-months post-PR** |
| **TIMEPOINT** | ***-t1*** | ***T_0_*** | ***T_1_*** | ***T_2_*** | ***T_3_*** |
| **ENROLMENT:** |  |  |  |  |  |
| **Eligibility screen** | X |  |  |  |  |
| **Informed consent** | X |  |  |  |  |
| **INTERVENTIONS:** |  |  |  |  |  |
| ***Pulmonary rehabilitationl*** |  |  |  |  |  |
| **Outcomes:** |  |  |  |  |  |
| ***Sociodemographic, anthropometric and general clinical data questionnaire*** |  | X | X | X | X |
| ***Health-related quality of life*** |  | X | X | X | X |
| ***Nº acute exacerbations*** |  | X | X | X | X |
| ***Healthcare utilisation and collateral costs*** |  | X | X | X | X |
| ***Symptoms*** |  | X | X | X | X |
| ***Impact of the disease*** |  | X | X | X | X |
| ***Emotional status*** |  | X | X | X | X |
| ***Peripheral muscle strength*** |  | X | X | X | X |
| ***Respiratory muscle strength*** |  | X | X |  |  |
| ***Exercise capacity*** |  | X | X | X | X |
| ***Balance*** |  | X | X |  |  |
| ***Physical activity*** |  | X | X | X | X |
| ***Peripheral muscle thickness*** |  | X | X |  |  |
| ***Cross-sectional area*** |  | X | X |  |  |
| ***Echointensity*** |  | X | X |  |  |
| ***Excursion and M-Mode Index of obstruction*** |  | X | X |  |  |
| ***Global rating of change scale*** |  | X | X |  |  |

**References:**

1. Chan AW, Tetzlaff JM, Gøtzsche PC, Altman DG, Mann H, Berlin JA, et al. SPIRIT 2013 explanation and elaboration: guidance for protocols of clinical trials. BMJ. 2013; 346: e7586.
